# Parents' Awareness of Bullying Involvement in Relation to Physician Practices: Implications for Practice Modifications

**DOI:** 10.7759/cureus.37301

**Published:** 2023-04-08

**Authors:** Tarika Nagi, Saurabh Somvanshi, Gautam Shanmuga Dharmar Balasubramania Pandian, Subbulakshmi Mohan, Satesh A Seegobin, Brian Altonen

**Affiliations:** 1 Psychiatry and Behavioral Sciences, Columbia University College of Physicians and Surgeons, Harlem Hospital Center, New York, USA; 2 Psychiatry, Ross University School of Medicine, Miramar, USA; 3 Data Analysis and Statistics, New York City Health and Hospital Corporation, New York, USA

**Keywords:** physician practice variability, psychiatry and mental health, pediatrics & neonatology, developmental and behavioural pediatrics, child and adolescent psychiatry, bullying and suicide

## Abstract

Background

Bullying is a complex abusive behavior with potentially serious consequences. Persons who bully and those who are bullied have consistently been found to have higher levels of depression, suicidal ideation, physical injury, distractibility, somatic problems, anxiety, poor self-esteem, and school absenteeism than those not involved with bullying.

Objectives

To our knowledge, no study has compared physicians’ practices of bullying prevention across different hospital settings and the effect of these practices on parents’ level of awareness. This article represents a subset (phase I) of the inter-departmental quality improvement study for comparing practices of healthcare professionals regarding bullying prevention between the pediatric outpatient clinic and child & adolescent psychiatry outpatient clinic, and parents’ awareness about provider’s anti-bullying practices.

Methods

Phase I was conducted as a cross-sectional study with the target population of adolescents (age 12-17 yrs) and corresponding guardians, seeking care from healthcare providers (residents, fellows and attendings) in the child & adolescent outpatient psychiatry clinic and pediatric outpatient clinic. It targeted both patients and providers, with adolescents/guardians completing questionnaire about bullying experiences, physician’s anti-bullying practices during past healthcare visits and adolescent Peer Relations Instrument. Providers answered questions about bullying assessing practices, level of self-preparedness and limitations.

Results

Data were analyzed in SAS 9.2 (SAS Institute Inc., Cary, NC) and SPSS (IBM Corp., Armonk, NY) and Chi-square tests were used for analyses of variables, and cross-comparing results for particular subsets. A total of 150 questionnaires were distributed. Among the provider surveys, self-reported level of preparedness (on a scale of 1-5; 1- least, 5-most) for assessing bullying was more in Psychiatry providers (Median 4, Mean 4.1) as compared to Pediatric providers (Median 3, Mean 2.9). In the first evaluation, very unprepared, unprepared and neutral (1, 2, 3) responses were contrasted with prepared to very prepared responses (4,5). The second evaluation excludes the neutral responses (3) and tests responses for the unprepared group (1,2) with the prepared group (4,5). The first evaluation resulted in Chi-Squared = 6.810, significant at p = 0.05 and the second evaluation resulted in Chi-squared = 4.774, also significant at p = 0.05.

Conclusions

This study identifies differences in healthcare professional’s anti-bullying practices and helps in identifying limiting factors. This identification of the practice gap helps in developing interventional strategies to improve the assessment of bullying situations across specialties.

## Introduction

Bullying is a complex abusive behavior with potentially serious consequences. Persons who bully and those who are bullied have consistently been found to have higher levels of depression, suicidal ideation, physical injury, distractibility, somatic problems, anxiety, poor self-esteem, and school absenteeism than those not involved with bullying [1-3].

Conflict vs bullying

There is a marked difference between conflict and bullying. Conflict and bullying are two distinct forms of interpersonal interaction that differ in several key aspects. While conflict can arise between individuals of similar power and status due to a difference in opinion or perspective, it is not characterized by an intention to harm or humiliate the other party. In contrast, bullying involves an imbalance of power where one individual holds a position of dominance over the other and engages in a persistent pattern of aggressive behavior toward the victim with the intention of causing harm or humiliation. Furthermore, bullying is often a systematic pattern of behavior, rather than a one-time event. The power differential in bullying can arise from factors such as physical size, age, social status, or position of authority. It is important to recognize the differences between conflict and bullying, as bullying can have severe consequences for the mental health and well-being of the victim.

To our knowledge, no study has compared physicians’ practices of bullying prevention across different hospital settings and effect of these practices on parents’ level of awareness [4]. This study represents phase 1 of inter-departmental quality improvement project for comparing practices of health care professionals regarding bullying prevention between the pediatric outpatient clinic and child & adolescent psychiatry outpatient clinic, parent’s awareness about bullying in relation to provider’s anti-bullying practices and identifies strategies for increasing parent’s awareness and healthcare professional’s expertise. This article was previously presented as an abstract at the 2017 AACAP 64th Annual Meeting on October 25, 2017.

## Materials and methods

Definition and its application

In 2014, the Centers for Disease Control and Prevention (CDC) and the Department of Education (DOE) jointly released the first federal uniform definition of bullying, aimed at providing a standardized framework for identifying and preventing bullying in schools and communities. The definition included three main components: unwanted aggressive behavior, observed or perceived power imbalance, and repetition of behaviors. Unwanted aggressive behavior refers to any intentional and harmful behavior, including physical, verbal, or relational aggression, directed toward another person. Observed or perceived power imbalance refers to an imbalance of power or control, which can occur based on factors such as age, size, strength, social status, or other characteristics. Repetition of behaviors refers to the persistent and ongoing nature of bullying, which may involve multiple incidents over time. The definition emphasizes the importance of considering the subjective experiences of those involved in a bullying situation, including the victim, the perpetrator, and any witnesses, and acknowledges that bullying can occur in various contexts, such as in-person or online. The adoption of a uniform definition of bullying by the federal government represents a critical step in addressing the issue of bullying and promoting a safe and supportive environment for all individuals. By providing a clear and comprehensive definition of bullying, educators, policymakers, and parents can better identify, prevent, and intervene in bullying situations, ultimately promoting healthy and positive relationships among children and adolescents.

Forms of bullying

Research has identified two main modes of bullying: direct and indirect [5]. Direct bullying involves overt aggression, such as physical violence, verbal abuse, and social exclusion, that is targeted at a specific individual [5]. Indirect bullying, on the other hand, is less visible and typically involves actions that are not communicated directly to the targeted youth, such as spreading rumors or engaging in covert forms of social exclusion [5]. To better understand the nature of bullying, researchers have identified four main types of bullying: verbal, physical, social, and cyberbullying [2]. Verbal bullying involves the use of hurtful language and derogatory comments, while physical bullying involves physical aggression, such as hitting, pushing, or kicking. Social bullying occurs when individuals use social power to isolate or exclude others, while cyberbullying involves the use of technology to intimidate or harass others.

Study protocol

The study protocols, informed consents, and data pooling procedures were approved by the institutional review boards (IRBs) of Health & Hospitals Corporation NY and Harlem Hospital.

Phase I was conducted as a cross-sectional observational study with a target population of adolescents (age 12-17 yrs) and corresponding guardians, seeking care from healthcare providers (residents, fellows, and attendings) in child & adolescent outpatient psychiatry clinic and pediatric outpatient clinic. It targeted both patients and providers, with adolescents/guardians completing questionnaire about bullying experiences, physicians’ anti-bullying practices during past healthcare visits, and adolescent Peer Relations Instrument. Providers answered questions about bullying assessing practices, level of self-preparedness, and limitations.

The survey targeted both clients and providers, with parents/guardians completing surveys about bullying experiences, physicians’ anti-bullying practices during past healthcare visits. Providers answered questions about bullying assessing practices, level of self-preparedness, and limitations.

## Results

Data were analyzed in SAS 9.2 (SAS Institute Inc., Cary, NC) and SPSS (IBM Corp., Armonk, NY), and Chi-square tests were used for analyses of variables, and cross-comparing results for particular subsets. A total of 150 questionnaires were distributed. Among the provider surveys, self-reported level of preparedness (on a scale of 1-5; 1-least, 5-most) for assessing bullying was more in Psychiatry providers (Median 4, Mean 4.1) as compared to Pediatric providers (Median 3, Mean 2.9). In the first evaluation, very unprepared, unprepared and neutral (1, 2, 3) responses were contrasted with prepared to very prepared responses (4,5). The second evaluation excludes the neutral responses (3) and tests responses for the unprepared group (1,2) with the prepared group (4,5). The first evaluation resulted in Chi-Squared = 6.810, significant at p = 0.05 and the second evaluation resulted in Chi-squared = 4.774, also significant at p = 0.05.

The Phase 1 study group consisted of 142 subjects comprising 101 clients (adult responders) with 50 clients being surveyed at the Department of Pediatrics and 51 clients at the Department of Child and Adolescent Psychiatry. The Provider population consisted of 41 clinicians (27 pediatricians and 14 psychiatrists). All analysis was conducted using the Chi-square test. Results greater than 3.841 at p=0.05 was considered significant as shown below in Table 1.

**Table 1:** Statistical Analysis of Physician and Patient Responses *Chi sq. >3.841 significant for p=0.05 #Assesses for only neutral or disagree responses, if response rates were significantly different. However, the number of responses <5 is enough to question statistical validity.

Physicians (n = 41)	Responses (n)	Outcome*	Chi Sq*
As a healthcare professional, how well prepared do you feel in assessing or screening your patients for their bullying involvement on a scale of 1-to-5?	41	Significant*	6.352
I screen my patients for bullying-related issues during their healthcare visits.	41	Significant*	6.470
If answer to 3 is Strongly Disagree, Disagree or Neutral, please check your reason. #	28	Not Significant	2.720
Patients (n = 101)			
My child’s healthcare provider has assessed my child for Bullying during his/her healthcare visits in past.	101	Not Significant	1.142
I was educated by my child’s healthcare provider on Bullying-related issues in children and adolescents.	101	Not Significant	0.848
I am interested in seeking information on the strategies to help end bullying situations and bullying prevention advice from my child’s healthcare provider during his/her healthcare visits.	99	Not Significant	0.835

**Figure 1:**
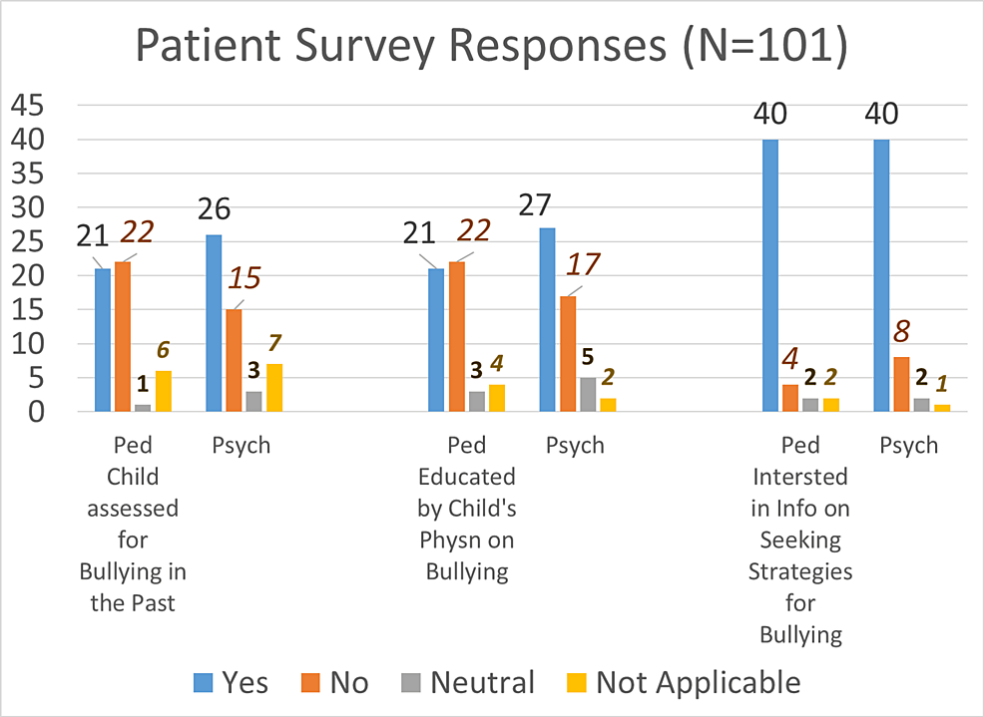
Patient Survey Responses

Among the client surveys, 51% of Psychiatry clients (versus 42% of Pediatric clients) reported being assessed for bullying during past visits and 53% of Psychiatry clients (versus 42% of Pediatric clients) reported being educated by their providers about recognizing symptoms of bullying in the clients. Both psychiatry (78%) and pediatric (80%) clients expressed interest in learning strategies to address bullying situations. The patient survey responses (Figure 1) were used to calculate the percentages.

**Figure 2:**
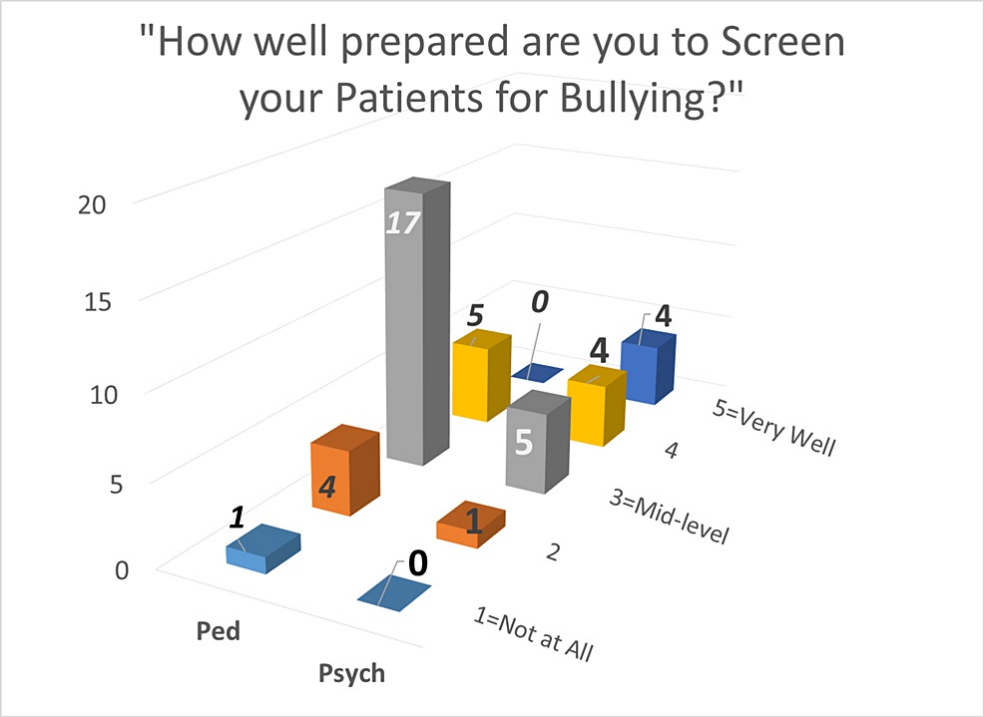
Preparedness to Screen Bullying

Among the provider surveys, self-reported level of preparedness (on a Likert scale of 1-5; 1- least, 5-most) for assessing bullying was skewed to higher levels of preparedness in Psychiatry providers as compared to Pediatric providers as shown below in Figure 2.

**Figure 3:**
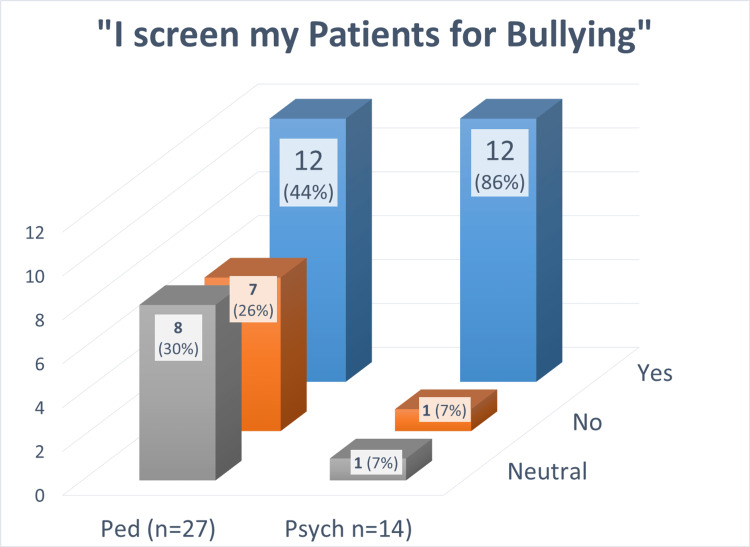
Pediatrics Vs Psychiatry Screening

The data was remarkable for the support for the hypothesized association between physician anti-bullying practices and physician specialty in that psychiatrists' screen children and adolescents more often for bullying in comparison to pediatricians in the outpatient setting. Based on the responses, only 44% percent of Pediatric providers reported screening clients for bullying compared with 86% of Psychiatry providers as shown below in Figure 3.

**Figure 4:**
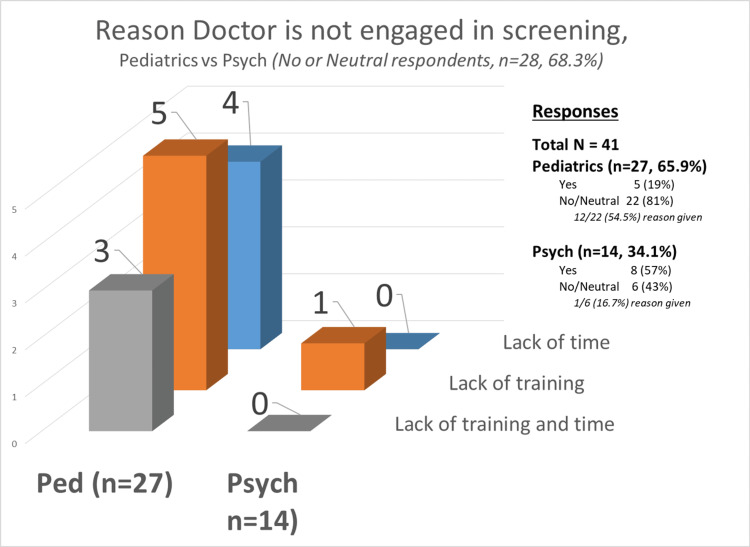
Reasons for not screening

Among the provider surveys, the major reasons reported for providers not being engaged in bullying screening were lack of training for psychiatrists whereas pediatricians also reported lack of time in significant numbers as shown below in Figure 4.

## Discussion

Effective management of bullying is a multidisciplinary effort, involving patients, parents, teachers, school officials, pediatricians, and mental health professionals. Where parents’ awareness and attitude are considered a key factor in preventing or controlling bullying behaviors and victimization, the role of healthcare professionals is also crucial in identifying at-risk patients and intervening in a timely manner [6,7]. By initiating a discussion about bullying during regular healthcare visits, healthcare professionals can increase the awareness about bullying in the community and provide appropriate intervention. However, different factors including lack of training or lack of time can interfere with this process. These findings highlight differences in healthcare professionals’ anti-bullying practices and help in identifying limiting factors as well as developing interventional strategies to improve the assessment of bullying situations across specialties. This is especially relevant to clinical trials focusing on prevention because, early to late adolescence is the developmental period during which social and role skills are consolidated, and it may be considerably more difficult to prevent adverse outcomes in individuals affected in this crucial developmental period. Improvement of the screening process, in itself, maybe a means of reducing the risk for later associated depression, suicidal ideation, physical injury, distractibility, somatic problems, anxiety, poor self-esteem, and school absenteeism than those not involved with bullying.

This study tested and found support for the hypothesized association between physician anti-bullying practices and physician specialty in that psychiatrists' screen children and adolescents more often for bullying in comparison to pediatricians in the outpatient setting. The reason for this observed difference was found to be a lack of training to perform these tasks, a lack of time to perform these tasks or both. It was found that there is almost twice as much published literature regarding bullying in the field of psychiatry as compared to the field of pediatrics which raises the concern that perhaps there needs to be more focus dedicated to bullying in the specialty. This study also highlights the importance and effectiveness of physician education with respect to preparedness to assess and screen for bullying in children and adolescent patients as one educational seminar increased the rates of self-reported preparedness from 'neutral' (2-3) to ‘very prepared’ (5).

Our study did not find a difference in parental awareness about bullying in relation to physician anti-bullying practices across pediatric and psychiatric clinical settings. It appears that the curiosity or desire to acquire information regarding bullying was independent of the differences observed in anti-bullying practices of both specialties. This could possibly be due to parental curiosity being more closely associated with their concern for their child’s wellbeing than it is related to the anti-bullying practices of their physician as this concern would remain consistent independent of the provider they are seeing.

There is a need to address the practice gap that is present in both pediatricians and psychiatrists with respect to identifying bullying involvement in their patients through targeted didactics and workshops for healthcare professionals. Distributing educational material on bullying and intervention guidelines to healthcare providers and encouraging them to use them during encounters with their patients may prove to be useful in both increasing the quality of physician anti-bullying practices and parental awareness of the nature and consequences of bullying for their child.

It is also important to note the critical role that a patient’s family plays in the health outcomes of a child experiencing bullying. One study looking at a group of eighty-one 6-year-olds in 1989 by Patterson et al. found that subjects whose interactions with their mothers were considered low in warmth and who were socially rejected by their peers at school were rated by teachers as having more behavioral problems and as less competent than other socially rejected students. Bowes et al. found that maternal and sibling warmth along with a positive environment at home promotes improved emotional and behavioral adjustment in bullied children compared to non-bullied children [8]. This study showed that warm family relationships and positive environments at the home act as protective factors buffering the child from negative outcomes associated with bullying victimization [9]. Implementing the standard in pediatric care to talk to parents about this routinely during healthcare visits may prove to have a significant positive effect over time on childhood resilience.

Pediatricians are the first line of contact for bullying intervention in the healthcare system. The American Academy of Pediatricians in 1999 issued a comprehensive policy statement outlining and defining the ‘emerging role of pediatricians in the prevention of youth violence’ but had not mentioned “bullying” in the report. A comprehensive analysis by Felitti et al. (1998) published a detailed investigation elaborating the role of adverse childhood experiences (ACE) brought bullying into acute focus after a series of high-profile, multiple-casualty, school-based shooting events took place in Pearl, Mississippi; West Paducah, Kentucky; Jonesboro, Arkansas; Springfield, Oregon; and Littleton, Colorado [10]. A reprint to this study was published in 2019 that included a section on bullying that specifically recommended the “promotion and reinforcement of parenting skills”, “recognition, screening, and appropriate referral as secondary prevention strategies”, and the use of the American Academy of Pediatrics (AAP) Connected Kids Violence Program [11]. Children with autism spectrum disorder (ASD) are bullied by peers at a rate three to four times that of nondisabled peers with negative impacts on academic functioning and mental health symptoms, including increased risk for suicidality [12]. While we know that the pandemic and its social isolation, loss of school experiences, increased screen use, and financial stress have likely had a psychological impact upon children and teens, little research has been done directly with youth to assess social and emotional factors during the pandemic and in its immediate aftermath. Female and LGBTQ youth were particularly vulnerable during the pandemic [13].

Our study highlights the need for and importance of anti-bullying practices of healthcare professionals in the primary care setting. Physicians have important roles in identifying at-risk patients, screening for psychiatric comorbidities, counseling families about the problem, and advocating for bullying prevention in their communities [14]. The findings reported suggest that a future direction should be to evaluate the extent to which adding bullying screening uniformly across medical disciplines may serve to improve the outcomes.

Strengths and limitations

This study has some methodological limitations, one of which is the size of the group studied. The number of participants involved in this study is at the lower end of that which is generally recommended for such studies but still within the range for statistical significance of p = 0.05. A slight modification of this study was made to ensure that the number of physician participants was closer to the number of parent participants. The Chi-squared evaluation of expected distributions confirms that this number can be relied upon. The literature search for article quantity in the field of psychiatry versus pediatrics also poses a possible limitation in that the over-representation in psychiatry may not be necessarily due to a lack of interest in the field of pediatrics, but that most severe symptomatic bullying cases that end up warranting further research are found due to some psychiatric intervention. It is also possible however that there needs to be further awareness of the implications of bullying in children in the specialty practice of pediatrics. Interpretation of these results should be done in light of these limitations. The educational presentation provided to physicians during their Grand Rounds meeting proved to have a significant effect on the self-reported preparedness of the physician to assess and screen for bullying. This measurement of physician preparedness may not prove to be the most accurate. A follow-up study measuring the number of patient visits where bullying assessment and screening took place before and after the educational intervention may prove to deliver more robust results highlighting the relationship between educational intervention and its effective impact on the practice of physicians.

The strengths of this study include the well-defined statistically matching patient populations. This suggests that any differences seen in the treatment process for these two groups pertain mostly to provider-related differences.

Future directions

We highly recommend that schools begin to systematically screen for adverse childhood experiences in their students - particularly those who are involved in bullying victimization or perpetration. Establishing this practice infrastructure in the educational system may prove effective in identifying patients at risk for developing long-term mental health and physiological issues. This screening practice could also reduce the number of interpersonal violent events that occur in the school setting. Further research needs to be done to develop the infrastructure in society to increase access to trauma-informed intervention services for students. A larger outpatient prospective study on bullying victimization would be of benefit to the current literature on this topic.

## Conclusions

To our knowledge, no study has compared physicians’ practices of bullying prevention across different hospital settings and the effect of these practices on parents’ level of awareness. Bullying victimization is a significant risk factor for suicidality in children and can lead to adverse educational, social, and health outcomes throughout adolescence into adulthood. There is much to be done to improve the healthcare system in such a way that it effectively identifies bullying events in school-aged patients to mitigate long-term consequences. This study highlights the importance of screening and assessing for bullying in school-aged patients. Physician barriers to screening can be resolved by implementing a brief question in routine healthcare visits such as “Do you ever get teased or picked on by other children?” or “Have other children tried to hurt you or bother you?” and proceed sequentially from there via prescribed guidelines.

A brief question implemented in a routine visit does not demand a significant time commitment nor does it require a significant amount of training. This question implemented in routine visits may very well be the item that increases the effective identification of suicidality and other mental health concerns in children and adolescents and is of crucial importance. There is a practice gap in physicians' screening of children and adolescents for bullying in a timely manner, failure to do so entails the risks for comorbid depression, anxiety, and suicidality that are implicated. With the knowledge of how significant bullying is in relation to healthcare outcomes, healthcare providers must solicit parental support through education and discussion and become an advocate for the patients in the school system and community.
